# Full Text and Figure Display Improves Bioscience Literature Search

**DOI:** 10.1371/journal.pone.0009619

**Published:** 2010-04-14

**Authors:** Anna Divoli, Michael A. Wooldridge, Marti A. Hearst

**Affiliations:** School of Information, University of California, Berkeley, California, United States of America; Northeastern University, United States of America

## Abstract

When reading bioscience journal articles, many researchers focus attention on the figures and their captions. This observation led to the development of the BioText literature search engine [1], a freely available Web-based application that allows biologists to search over the contents of Open Access Journals, and see figures from the articles displayed directly in the search results. This article presents a qualitative assessment of this system in the form of a usability study with 20 biologist participants using and commenting on the system. 19 out of 20 participants expressed a desire to use a bioscience literature search engine that displays articles' figures alongside the full text search results. 15 out of 20 participants said they would use a caption search and figure display interface either frequently or sometimes, while 4 said rarely and 1 said undecided. 10 out of 20 participants said they would use a tool for searching the text of tables and their captions either frequently or sometimes, while 7 said they would use it rarely if at all, 2 said they would never use it, and 1 was undecided. This study found evidence, supporting results of an earlier study, that bioscience literature search systems such as PubMed should show figures from articles alongside search results. It also found evidence that full text and captions should be searched along with the article title, metadata, and abstract. Finally, for a subset of users and information needs, allowing for explicit search within captions for figures and tables is a useful function, but it is not entirely clear how to cleanly integrate this within a more general literature search interface. Such a facility supports Open Access publishing efforts, as it requires access to full text of documents and the lifting of restrictions in order to show figures in the search interface.

## Introduction

The PubMed system from the National Library of Medicine (http://www.ncbi.nlm.nih.gov/pubmed/) is the primary tool used by biologists to search the literature. PubMed's interface has a number of useful features and popular innovations, most notably its facility for recommending articles related to a given article [2], [3], but currently search in PubMed is restricted to the title, abstract, and several kinds of metadata about the document.

On the Web, searching within the full text of documents has been standard for more than a decade, and much progress has been made on how to do this well. However, until recently, full text search of bio-science journal articles was not possible due to constraints on online availability and intellectual property restrictions. Recent developments in the opening up of the content of journal articles (including requirements for open access publishing by national funding agencies in both the U.S. and the U.K.) allow for improvements in the design of such interfaces.

It should be noted that freely accessible articles are not necessarily articles that can be crawled, stored, and indexed by researchers. Only the small subset found in the PubMedCentral Open Access collection of journals provides an unrestricted resource for scientists to experiment with for providing full text search (the license terms for PubMedCentral can be found at: http://www.pubmedcentral.gov/about/openftlist.html).

Full text availability allows for a re-thinking of how search should be done on bioscience journal articles. For instance, many researchers are using full text biology articles for information extraction (text mining), as seen in the BioCreative competition [4], [5]. The results of text extraction can then be exposed in search interfaces, as done in systems like iHOP [6] and ChiliBot [7] (although both of these search only over abstracts).

Another question is how to adjust search ranking algorithms when using full text journal articles. For example, there is evidence that bioscience literature ranking should consider which section of an article the query terms are found in, and assign different weights to different sections for different query types [8], as seen in the TREC 2006 Genomics Track [9].

Another way to innovate with full text article search is to specialize the interface to correspond to the particular needs of a particular field or collection. For the last several years, Google Scholar (http://scholar.google.com) has provided search over the full text of journal articles from a wide range of fields, but with no special consideration for the needs of bioscience researchers. Google Scholar's distinguishing characteristic is its ability to show the papers that cite a given article, and rank papers by this citation count. This is an excellent feature for journal article search, and all such systems should use citation count as a metric. Unfortunately, citation count requires access to the entire collection of articles; something that is currently only available to a search system that has entered into contracts with all of the journal publishers.

This article focuses on another way to improve bioscience literature search: provide the user with the ability to search over full text, including figure captions, and display the associated figures alongside search results. This idea is based on the common observation that researchers, when reading bioscience articles, tend to start by looking at the title, abstract, figures, and captions. Figure captions can be especially useful for locating information about experimental results [10].

**Figure 1:**
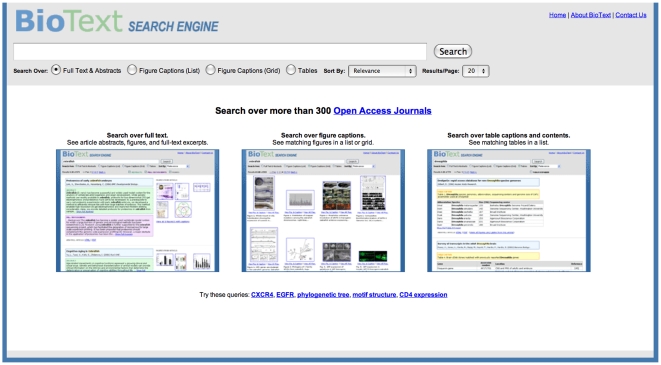
BioText Search: home page and query form. The home page and query form for the BioText Search Engine, as used in the usability study described in this article. To help ease users into a novel search interface, it can be useful to provide hints about how the interface works in a simple manner on the home page of the website. The BioText search engine employs two such techniques. The home page shows links to sample queries as a low-effort way to entice users who are unsure how to start into interacting with the system. The home page also provides reduced-size renderings of three different search results views. At the top of each view within the interface appears the query entry form. It provides: 1. Links to information about the search engine and the collection it indexes. 2. The entry form for entering the query, along with a search button (hitting Return on the keyboard is equivalent to selecting this button). 3. A radio button for selecting among the different subcollections and views (full text with figure view, table text with table view, etc). 4. A drop-down menu selector for choosing the search results ordering (one of Relevance, Descending Date, Ascending Date). 5. A label indicating the number of retrieved documents, and hyperlinks allowing the user to select which page of results to view. Additionally, the full text with figure display view shows a set of checkboxes that dynamically determine which parts of the documents surrogates to show or omit (abstract, full text excerpts, and figures). By default, all three are selected, but if the user unselects any of the checkboxes, that selection is retained into subsequent searches. The footer of each search results page also shows the total number of hits, along with the selector for other pages of hits. At the time of the study, selecting a radio button did not automatically re-run the query and change the results view; rather, the user had to select the Search button to activate the change (as noted in the study results below, the automated behavior is expected).

Our research group has developed a freely available search interface called BioText (Figure 1 and http://biosearch.berkeley.edu) for searching the literature and showing figures alongside search results [1]. We conducted a pilot study exploring user reaction to searching caption text and incorporating figure display into a bioscience literature search interface [11]. The participants in that study had strong positive reactions to the idea: 7 out of 8 said they would use a search system with this kind of feature if all of the literature were available in the collection. That study also found that participants were interested in searching the full text, not just the captions, and that some were interested in searching table text as well.

The study presented here is a follow-up to the pilot study, using a larger number of participants and incorporating a number of improvements and extensions to the interface, including a facility to search the full text (as opposed to searching only the abstract and caption text). This study was qualitative; 20 participants were asked to use the different views of the interface and response to it in terms of how likely they would be to use it, which aspects they did and did not like, and which missing features would they like to see added.

### Related Work

#### Analyzing caption text and linking it to figure content

Several research projects have examined the automated analysis of text from captions. Srihari [12], [13] did early work on linking information between photographs and their captions, to determine, for example, which person's face in a newspaper photograph corresponded to which name in the caption. Shatkay et al. [14] combined information from images as well as captions to enhance a text categorization algorithm.

Cohen, Murphy, Qian et al. have explored several different aspects of biological text caption analysis [15], including algorithms for parsing the structure of image captions, and techniques for extracting information relating to subcellular localization by automatically analyzing fluorescence microscope images of cells [16], [17].

Liu et al. [18] collected a set of figures and classified them according to whether or not they depicted schematic representations of protein interactions. They then allowed users to search for a gene name within the figure caption, returning only those figures that fit within the one class (protein interaction schematics) and contained the gene name.

Yu et al. [19] created a bioscience image taxonomy (consisting of Gel-Image, Graph, Image-of-Thing, Mix, Model, and Table) and used Support Vector Machines to classify the figures, using properties of both the textual captions and the images. Yu and Lee [20] developed algorithms to link sentences from an abstract to the figure caption content. They also developed and assessed a user interface called BioEx that shows a set of very small image thumbnails beneath each abstract, but the system described did not allow for searching over text corresponding to the figure caption and did not focus how to design a full text and caption search system in general.

More recently, Yu et al. [21] asked human judges to determine how much of the important information about a figure was present in the figure caption vs. the abstract and full text of the article. They found that having access to caption, title, and abstract alone led to less complete comprehension than having the full text of the article available.

#### Analyzing figure content

Several approaches have processed the images themselves to extract meaningful information to use in search results. Christiansen et al. [22] used automatically-computed properties about the content of raster images within an article in order to improve relevance feedback techniques for the literature review task. That work also examined how to associate the caption text with the appropriate image when processing PDF documents. Deserno et al. [23] examined the potential impact of performing content analysis on the images in the figures in order to improve literature retrieval. In the Yale Image Finder, Xu et al. [24] developed algorithms to extract text strings from figures and provided a figure searching tool that queries over the extracted figure text as well as caption text. In addition to the figure containing the query term hits, the other articles from the same paper are shown as well as figures from other papers with similar image content.

#### Showing figures in Web search results

On the Web, for information-centric queries, it has been shown that richer results listings can be more useful and preferred over short snippets [25], [26]. However, efforts to use thumbnails of web pages have not been particularly successful at improving search results using standard metrics. A study by Czerwinski et al. [27] showed that after a brief learning period, blank squares were just as effective for search results as thumbnails, although the sub jective ratings for thumbnails were high. A subsequent study by Dziadosz et al. [28] found that thumbnails alone were much more error-prone than the other two conditions; also, the number of errors in text alone versus text plus thumbnails was nearly identical. Additionally, showing thumbnails alongside the text made the participants much more likely to assume the document was relevant (whether in fact it was or not). On the other hand, in some studies, the thumbnails may have been too small to be effective. Kaasten et al. [29] systematically varied the sizes of web page thumbnails shown, and found participants were able to more accurately recognize web pages when larger thumbnails were shown in combination with titles, than with titles alone. When thumbnails were smaller, participants relied on color and layout to recognize the page, and could only make out text at larger image sizes. Kaasten et al. [29] also found that in their study, 61% of the time, thumbnails were seen as very good or good representations of the underlying web page, and 86% were very good, good, or satisfactory.

One problem with using thumbnails is that they create an image from an entire page, which can end up showing only miniaturized text. By contrast, BioText uses figures extracted from articles. Although some figures from bioscience articles are not particularly distinguishing, in many cases the general information visible in the figures distinguishes the kind of information contained in the article. For instance, the figures associated with articles that are retrieved in response to a query on “lung” range from x-rays to histology images to schematic diagrams to flow charts to line graphs, and this kind of information can be highly indicative of whether or not the article is of interest to the scientist.

### The Interface Used in This Study

The BioText search engine indexes all Open Access articles available at PubMedCentral. To date, this collection contains more than 300 journals, 129,000 articles, 247,000 figures, and 104,000 tables. A script is run on a daily basis that checks the Open Access database for updates and adds new documents to the collection.

**Figure 2:**
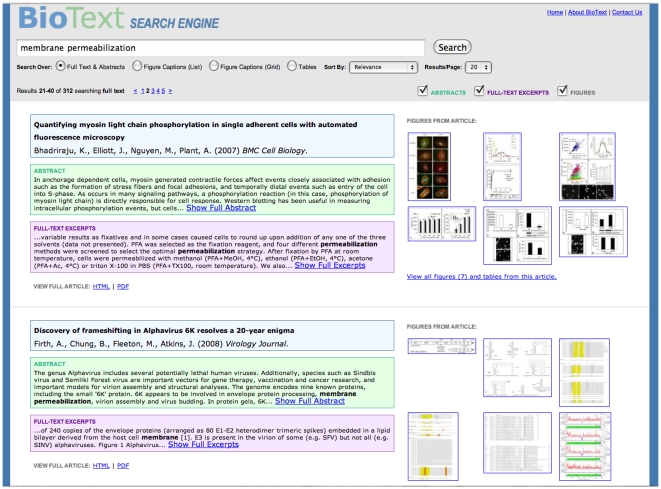
BioText Search: full text search results with figure display view. The full text search with figure display searches over, and consecutively displays, the article's title, authors, abstract, and the full text from the body of the article. This is the primary (and default) view for the system. For each retrieved document, a display is shown on the left-hand side that consists of a vertical list of textual information from the article. This list consists of the document's metadata (title, authors, journal, publication date), the document's abstract, and excerpts from the full text of the document that contain query term hits. Additionally, for each retrieved document, small thumbnail versions of the first six figures from that article are shown on the right-hand side of the display, along with a link to see the document summary view labeled “View all K figures and captions” where K is the actual number of figures found in the document. Clicking on the figure itself produces a new page that shows the full-size figure along with its caption text. Throughout the entire BioText interface, each text area type is assigned a background color, and this color is kept consistent throughout the interface (e.g., yellow is the background color for caption text in all of the interface views). As mentioned above, the user can choose to reduce the amount of information displayed, either for the current query or for the entire search session. For example, the user can choose to view only titles and figures. Additionally, if a text area exceeds a predefined length threshold (approximately 500 characters), the text is cut off, and an ellipsis is shown along with a link to “Show Full Abstract” or “Show Full Excerpts.” If the user clicks on the link, the text is expanded in place, and at the end of the text a link is shown that allows the user to reverse the procedure (“Shorten Abstract” or “Shorten Excerpts”). Query terms are highlighted in the text areas via boldface font. In the title area, the title text is all in boldface, and so a yellow highlight background is applied to the query term hits within the title. In the full text as well as all the other search results views, a hyperlink is shown that allows the user to view the article directly as HTML or PDF, or in the summary version provided on the BioText site.

**Figure 3 pone-0009619-g003:**
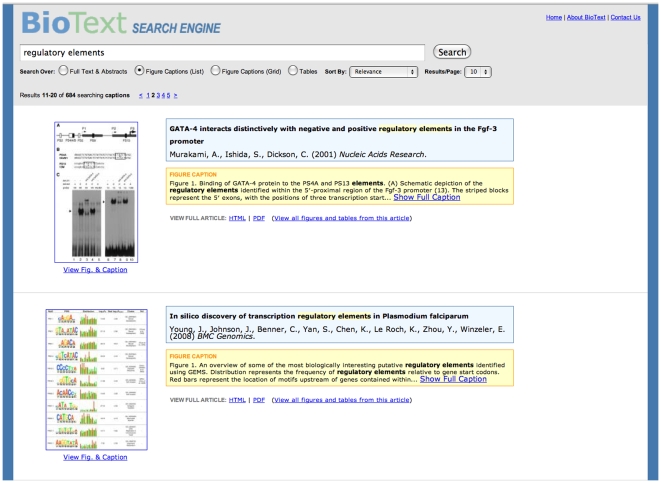
BioText Search: caption text search results with vertical list figure display view. The figure captions list view shows the results of searching over the text of the article's title, the authors, and the figure captions, so the items retrieved can differ from that of full text figure view (Figure 2). The figure for each document is shown as a larger-sized thumbnail (compared to the full text view) and is shown to the right of the title and caption text, to emphasize the difference with the full text figure view. It was thought it would be important to signal this difference because the search results differ when searching over caption versus full text. As in the full text search view, clicking on the figure's thumbnail shows the full size image along with its caption in a new window.

**Figure 4 pone-0009619-g004:**
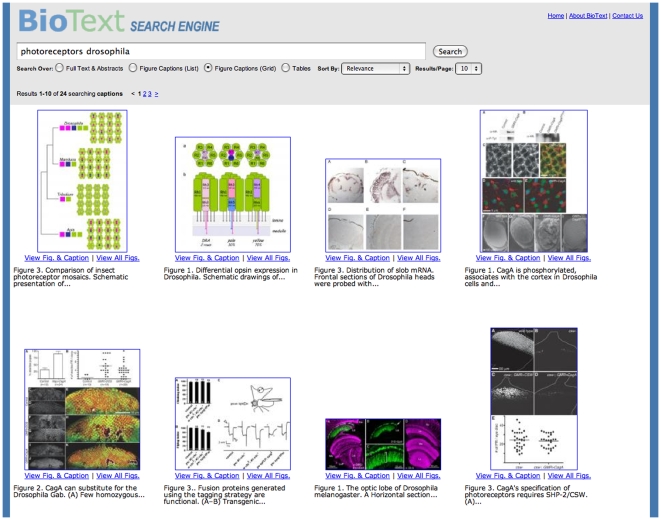
BioText Search: caption text search results with grid figure display view. The figure captions search and figure grid view searches in the same manner as in the figure captions list view (Figure 3), but shows the matching figures as thumbnails arranged in a 4×5 grid layout together with some citation information. This view is intended to be similar to that of web image search interfaces. Beneath each figure is shown a link to view the full-sized figure and its caption and another link to go to the article summary view. The first 100 (approximately) characters of the caption are shown, followed by an ellipsis. A mouse hover over the figure shows more complete metadata about the figure, namely its title, authors, journal and publication date.

**Figure 5:**
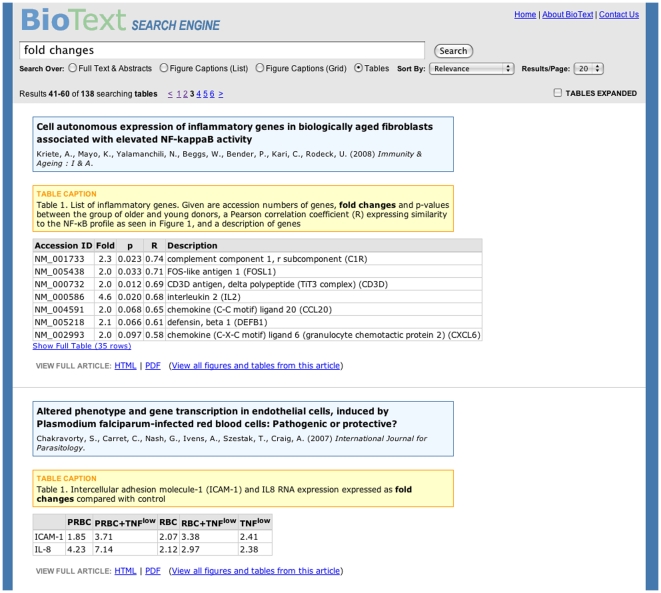
BioText Search: table caption and text search results with table display view. The table view searches over the text within tables as well as the table captions and the corresponding articles' titles. The matching tables are displayed together with their captions and the title, authors and citation of the article they originate from. The results are shown as a vertical list consisting of the article's title and other metadata followed by the caption followed by an HTML rendering of the table, so that all tables in the interface have one consistent appearance. If more than one table occurs within a given article, the other information is currently repeated for each table. Tables longer than 8 rows long are truncated and a link is shown that allows the user to expand the table to its full length. Additionally, a checkbox is shown in the query form area that allows the user to see all tables fully expanded; the default is for this to be unselected.

**Figure 6:**
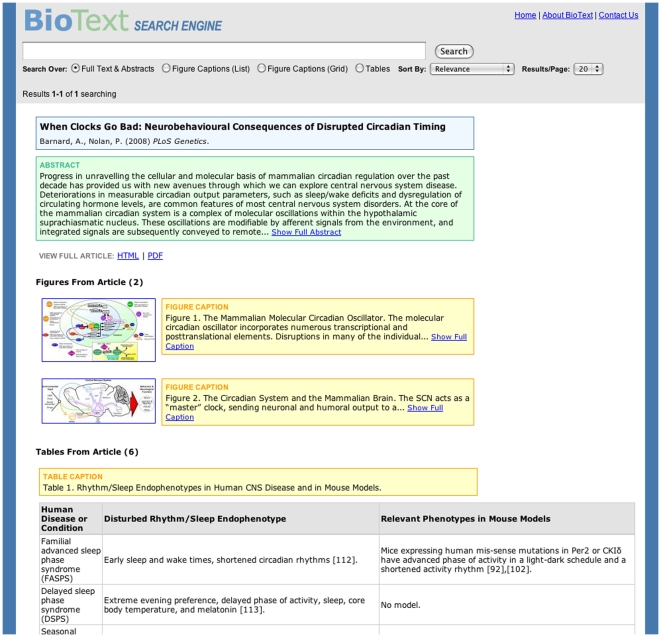
BioText Search: summary document view. In each of the four results views (Figures 2–5), the user can click on a link that brings up the full text of the paper, either in HTML or PDF format (there is no need to first go through PubMed because the collection consists entirely of Open Access documents). Alternatively, the user can select a link labeled “View All Figures and Tables from this Article,” which is shown alongside search hits. This produces a view in HTML showing the article title (along with authors, journal, and date), abstract, and a vertical display view of all of the figures and tables alongside their captions. During the study, there was a problem with this view in that it retained the query form at the top of the page but removed the query that produced the search results listing that led to the article. A better solution is either to remove the query form from this view, or provide a link back to the search results for the previously issued query.

The main components of the interface are:

- Home (Starting) Page and Query Form (Figure 1)
- Full Text with Figure Display (Figure 2)
- Caption Text with Vertical List Figure Display (Figure 3)
- Caption Text with Figure Grid Display (Figure 4)
- Table and Caption Text with Table Display (Figure 5)
- Detailed Article Summary View (Figure 6)

The main innovation of the BioText search interface that distinguishes it from PubMed and other bioscience literature search interfaces is its emphasis on showing the figures associated with the articles within the search results, and showing the full text context in which the query terms fall within the article, including the captions. (The version of the interface presented in [11] searched only over caption text, and the version in [1] searched only over abstracts and captions.)

Considerable effort went into the design of the display of the sections, to enhance legibility and reduce impressions of clutter. These design decisions surrounded choices in the relative font sizes, the font types, the heaviness of the border lines in the boxes surrounding each section, and the spacing between the text boxes and the figure displays. For instance, the labels for each text area (e.g., “Full-Text Excerpts”) is shown as small-caps in a more saturated color than the area background. The design was modified and evaluated among the authors over several iterations.

The current search interface uses what are known as search “verticals” for showing different views of the search results. Results from search engine literature suggest that most users do not switch away from the default view into verticals, and the major search engines are moving to “blended” or “universal” search in which hits from different parts of the vertical space are interwoven with one another and act as an entree into the more specific type of search.

For instance, a search on baseball at Google yields standard links to web pages but also a set of links to image results, with a hyperlink labeled “Image results for baseball”. This link moves the user into the image search vertical and raises their awareness of this option that is available from the site [30]. Blended results might be a good way to introduce users to alternative views of search results, especially for the Figure Grid Display view. But for experimenting with the different views explicitly as done in this study, it is useful to retain the differentiation between the views.

Open access journal collections such as Highwire Press and PubMedCentral allow for search over the full text, but the search results listings only show where the hits fall within the title and abstract. PubMedCentral furthermore provide a facility, under its “Limits” option, to choose “search over figure/table caption” from a long list of options. However, the search results display does not show the caption text, nor do they show the corresponding figures or tables.

As discussed above, Google Scholar searches the full text of documents but only displays a very brief snippet of content for the search hit. BioText emphasizes the display of a richer document surrogate, thus facilitating the assessment of the relevance of a document directly within the retrieval results. This richer information is not necessary for all search tasks (for example, when searching for the home page of a website, a shorter surrogate is better), but has been shown to be useful for information-intensive tasks [25]. Thus for a researcher who is scanning the contents of a journal to see what's new, the richer information may not be helpful, but for a researcher who wants to understand how an experimental method is used, this extra information can be quite useful, as seen in the results below.

### Implementation Details

The Lucene search engine (http://lucene.apache.org) is used to index, retrieve, and rank the documents, using Lucene's default statistical ranking functionality.

The articles are downloaded in XML format, and a script is run to separate out the different parts of the document. The figures are stored locally, and at different scales, in order to be able to present thumbnails quickly. The tables are also stored locally. Lucene's indexing facility is used to index several fields separately, including title, authors, abstract, caption text, full text, and table text. Lucene allows the user to assign different weights to these fields for use in the statistical ranking algorithm. For the Full text index (used in the Full Text Image Display View), the text in the title and abstract is assigned higher weight than the text in the rest of the article. This means that articles in which the query terms appear in the title and abstract are ranked higher than those in which the query terms appear only in the full text. Specifically, the relative weights for the Full Text with Figure Display index are:

- Title - 5.0
- Full text - 0.1
- Abstract - 2.0
- Authors - 1.0

These weights were arrived at by trial and error; users disliked a ranking in which articles without hits in the title and abstract were ranked higher than those with such hits. Ideally, a weighting would learned via machine learning over a large dataset of user behavior, as is currently done for commercial web search engines [31]. (Comparing different section weighting algorithms would be a topic for a separate paper.)

For the Caption Text indexes (both Vertical List view and Grid view), all fields are weighted equally:

- Title - 1.0
- Caption - 1.0
- Authors - 1.0

For the Table Text and Caption index, the weight on terms from the table caption is increased, text from author names is not included in the ranking:

- Title - 1.0
- Table Text - 1.0
- Table Caption - 2.0

Additionally, for the Full Text with Figure Display view, the Lucene Highlights package is used to extract excerpts from the full text of the retrieved documents, to extract up to six passages that contain query term hits, and to highlight the query term hits within the excerpts.

The interface presented to the user is a combination of HTML and javascript. The interface is generated by code written in Python, Perl and PHP. Logs and other auxiliary information are stored in a MySQL database.

## Results and Discussion

### Hypotheses and Results

As mentioned above, based on the conclusions of our pilot study [11], we made several modifications to the interface. Those conclusions also led to the following hypotheses, which we investigated in the study reported here:

- H1: Most participants would have a favorable response to the display of the article's figures next to the search results, for most information seeking tasks.
- H2: Most participants would have a favorable response to searching over the full text, as opposed to just the abstract and title, for the primary search view.
- H3: Some participants would find the grid view with caption search appealing for specialized information seeking tasks.
- H4: Some participants would find the table view with table text and caption search appealing for specialized information seeking tasks.

Hypotheses H1 and H2 are supported, as shown in Table 1. Participants were asked to state, at the end of the study session, how often they would be likely to use each interface view, assuming all of the bioscience literature were in the collection. 19 out of 20 participants said they would use the full text search with figure display interface either frequently or sometimes.

**Table 1:** Participant responses to different interface views.

Response	Full Text Search	Figure Caption Search	Table Search
Frequently	15	8	6
Sometimes	4	7	4
Rarely	0	4	7
Never	0	0	2
Undecided	1	1	1

Number of participants who rated each view according to how often they thought they would use that view.

However, participants were not asked to explicitly state if the reason for this preference is the showing of the figures or the search over full text, or both. Given that, further support for H1 is that, when asked to comment in more detail about which aspects they did and did not like about each view, 11 participants volunteered that they liked to see the figure thumbnails, and no participants stated that they disliked seeing the figures, although 5 people said the default view should be only titles and metadata. Further support for H2 is that 5 participants explicitly stated that they liked being able to see the text excerpts, 2 stated they liked seeing the variety of information within the search results listing, and no participants stated that they disliked the full text search (again, keeping in mind that 5 said the default view should be only showing title and metadata information). Thus, as seen in “blended” results in web search, many participants in this study liked the combined output view.

Additional comments about the full text figure display view, along with the number of participants who mentioned these points, are shown in Table 2. Notable and frequent among the favorable comments are mentions of the intuitiveness, clarity, and compactness of the design and the layout, including numerous mentions of the use of color and query term highlighting. Unfavorable aspects included a subset of people who preferred less information by default and some issues about size of the screen and the figures.

**Table 2:** Detailed responses to full text search with figure display.

Text View – Favorable Aspects	
11	Ability to see figure thumbnails.
7	Direct links to full paper without going through PubMed.
5	Ability to see excerpts.
5	Colors are helpful.
5	The layout – it is easy to navigate.
5	Highlighting.
4	The expand options don't require reload so it is fast.
3	Clear.
2	Variety of info at once the parts that people read first in papers.
2	Compact/easy to browse.
2	Intuitive/simple.
2	Option to select what to display abstract, excerpts, thumbnails.
1	The narrow horizontal width of title and abstract – more readable.
1	Not distracting despite all the information.

Text View – Unfavorable aspects	
5	By default should display titles only.
3	When unselecting abstracts, figures and full-text, so you can browse quickly through titles, it would be good to have the option to open individually just one abstract individually, rather than all or nothing.
2	Given that the system shows figure thumbnails, it should show tables too.
2	Should see all thumbnails, not limited to six.
2	Should use different colors especially the yellow.
2	Should provide better citation display, especially journals, and a more precise date.
1	When “going back” from the endgame view to the search results, should go to the part of the page you were and not back at the top stated for all view types.
1	Descending date should be the sorting default.
1	The screen is too wide – should be resizable.
1	Thumbnails should be bigger.
1	Should display relevance score when sorted by relevance.
1	The color of the purple boxes jumps out too much.

Text View – Requested Additional Features	
2	Should provide function for users to select hits they like.
2	Should provide a link to supplementary data and DOI along with PDF/HTML
1	Should highlight the author name in the citation and should allow author search.
1	Provide function to click on author name and show all hits from that author.
1	Provide ability to export to EndNote.
1	Should show total count of hits for the query appearing in the full-text and in which sections.
1	Should have highlighted the query terms in the HTML version of the full paper notjust in the search results; note that the HTML is hosted by the journal provider.

Comments from participants about the Full Text with Figure Display View, paraphrased, after using all of the interface options and then revisiting the full text view.

Moving on to H3, 15 out of 20 participants said they would use one of the two caption search and figure display interfaces frequently or sometimes. Table 3 shows some of the detailed likes and dislikes as well as feature requests for these views. As seen in our pilot study, there are differences of opinion as to which of the caption view interfaces (vertical list or grid) is better. This difference seems to hinge on how much caption information is visible within each view. A suggested feature was to support the grouping of all figures and captions from one paper together in the figure views (ignoring the relevance ranking of the caption for the query).

**Table 3:** Detailed responses to caption search with figure display.

Figure Views – Favorable Aspects	
5	Ability to search in captions.
5	It is good to have two figure view options; grid is good for a quick browse.
3	Clear display/layout.
3	The caption is viewable without extra work.
2	Colors are easy to keep track what you are looking at.
2	Pop-up title in grid view.
2	Highlighting.
2	Compact.
2	Good for when you look for an image.
1	Everything.
1	Direct links to full paper.
1	Clicking on figures open in new window.
1	Ability to link to all figures from one paper.

Figure Views – Unfavorable Aspects	
3	Figures should be on the right to match the text view layout.
2	Figures from the same paper should be under the same title.
2	Alongside each figure, provide a way to see or jump to other figures from the same
1	Figures should be displayed in the order they appear within a paper, if more than one figure from a given paper occurs in the results.
1	Preferable to have newest figures first.
1	The color yellow of the caption jumps out.
1	Bold is good for highlighting but not the yellow.
1	Remove grid view, it is redundant.

Figure Views – Additional Requested Features	
1	Should have link to full papers from the grid view.
1	Should support author search – search in author field.
1	The title box should be yellow.
1	Should be able to search on all fields not just captions from this view.
1	When a figure opens in a new window should have citation info and links to full-text PDF/HTML.

Comments from participants about the Caption Search with Figure Display both Vertical List and Grid View, paraphrased, after using all of the interface options and then revisiting the caption search views.

As for H4, table search view was seen as less generally useful, with only 10 out of 20 saying they would use it frequently or sometimes, but with another 7 saying they would use it rarely, suggesting that it is anticipated to be useful only for specialized information needs. Table 4 shows other comments made specifically about the table view. Some participants liked that the table view gave them the opportunity to look for information that can be found in tables but nowhere else in the paper, especially for experimental results.

**Table 4:** Detailed responses to table text and caption search with table display.

Table View – Favorable Aspects	
5	Access to information that is in tables but nowhere else.
4	Useful, good for finding information.
4	By default the tables are short.
4	Expand table length option.
4	Clear.
4	Standardized, all tables in same format not as they appear in papers makes it easy to browse.
3	Highlighting.
2	Informative to have title and caption with each table.
1	Colors.
1	Direct link to full text.

Table View – Unfavorable Aspects	
5	Table display is not as useful/informative as images when shown in isolation because context is missing.
4	Should have different highlighting inside the tables, a way to stand out more.
3	Caption color too strong and confusing with the yellow highlighting.
1	In the non-expanded mode, would be good to see the part of the table that the query was found, not the top few rows, but still see the header of the table.
1	Tables from the same paper should be under one entry/title.
1	Would be better for tables to open in new window rather than expanding on same page.
1	Would prefer caption under the table–more traditional.

Comments from participants about the Table Caption and Text Search with Table Display, paraphrased, after using all of the interface options and then revisiting the table search view.

Participants were also asked to estimate how often they would use the different views for different types of tasks. Table 5 shows how often participants responded “frequently” or “sometimes” for each view type although it may be more difficult for people to accurately estimate usage at this fine-grained a level. (Because not every participant is interested in every type of task, only some answered the question for given task types.) When broken down by task type, table caption search and display were seen as potentially useful for certain tasks, such as finding new developments and learning about experimental methods.

**Table 5:** Estimates of use of search results views for specific task types.

Type of Information Need	# Responses	Full Text	Figure Caption	Table Caption
Related Papers	10	10	8	4
Specific Information	10	10	8	6
Background Information	15	15	10	6
Reviews	8	8	4	1
New Developments/Findings	20	20	13	10
Experimental Methods	19	18	9	8

Estimates by participants of whether they would use the different views for different types of tasks. (Because not every participant is interested in every type of task, only some answered the question for given task types.) Numbers indicated how many participants responded “frequently” or “sometimes” for each view type.

### Other Observations

Across the views, participants requested features that are outside the goals of this study but are often present in literature search engines. Two participants felt strongly enough about their way of searching the literature to describe their methods in detail. Two use email alerts to save time on looking up for updates in their areas of interest. Seven participants mentioned scanning titles as a fast and effective way to detect interesting literature. Author and journal names were also used during the scanning process. Display of accurate date was also important to one participant, to be sure of what is the latest news in their field (the interface currently displays and sorts by year, not by month and day).

Regarding the BioText interface, 11 participants mentioned, in a variety of ways, that they liked the convenience of going directly to the figures, and to the full text of the papers, without having to go through PubMed or an intermediating journal site. Participants also liked having the larger versions of the figures, along with their captions, shown in a new window, but two mentioned that the title and other citation information should be shown in addition.

Two participants said that they liked the table view because tables often summarize results in papers, and appreciated that each table was shown with the corresponding caption and title. However, two participants felt that the context was not enough when a table is isolated from the full paper. As with figures, some participants asked that tables from the same paper be grouped together, independent of the relevancy ranking scores. Two participants requested that the system find a way to display abbreviated versions of the tables along with the figure thumbnails in the full text search results screen, although it is unclear how to useful a table “thumbnail” would be.

Generally, participants were not aware of many of PubMed's features (including its support for Boolean queries). This finding is not surprising, because few people are trained in its use and the advanced features are hidden behind drop-down menus which are less likely to be experimented with. We told all the participants that the collection was small because it contained only Open Access articles. Two participants went out of their way to ask why our collection was not larger, and were surprised to hear about the difference between free and Open Access articles.

### Usability Studies In Biomedicine

Usability studies serve as a valuable evaluation measure for search engines and influence the design of all commercial search systems. As biology is becoming an information science, numerous tools with search facilities have been made available to bioscientists, but unfortunately not many usability studies have been performed/published that investigate the idiosyncrasies of search and design needs in biology.

The BioText system offers a great platform for investigating how to display the different components of full biomedical journal papers to users according to their information needs. More such studies need to be published to get a better understanding of the bioscientists search preferences. There is a number of standard methods used to evaluate search interfaces [32], depending the aim of each study. There are informal usability studies that help determine the design of a search interface, formal studies (control experiments) that help determine how specific design elements work for certain tasks, longitudinal studies that allow to understand users behavior over time and other large scale and log analyses that allow studying how current users react to variations of the interface. Such findings would benefit the whole text mining community, providing better insight on how to design systems that aim to end users.

### The Need for Full Open Access Publishing

Since most participants named PubMed as their literature main search system, the results presented in this paper suggest that the study participants would prefer the kind of functionality provided by BioText to be present in PubMed, but it is not entirely clear how to cleanly integrate BioText's features within a more general literature search interface. In order to determine for certain if this kind of interface is superior to standard search, thousands of users need to use the site. An analysis of the server logs of the search engine found that starting from launch in September 2007, and analyzing up through November 2008, there were nearly 24,000 queries and views of results pages, originating from approximately 11,000 unique IP addresses. However, indexing only a subset of the literature is unacceptable for widespread use. In this study we had to ask participants to pretend that the system indexed everything in MEDLINE, but in order to make a truly competing interface, all articles must be present, as users blame the interface if they cannot find the documents they expect, and many of the high impact journals are missing from the Open Access collection. (A fully competitive interface also needs to provide other services such as citation linking, which also requires access to the full text.) Thus the results of this research cannot be fully tested until full text of all (recent) research articles is made freely available for search interfaces.

### Conclusions

This study found strong support for the hypothesis that bioscience literature search systems such as PubMed should show figures from the articles alongside the search results. Additionally, it found evidence that full text and captions should be searched along with the article title, metadata, and abstract. Finally, for a subset of users, allowing for explicit search within captions for figures and tables is a useful function. We hope that with further support by the scientific community of the Open Access publishing model, more biomedical literature systems will take advantage of full text, figures, tables, and their captions, in their searches.

## Methods

The goal of the BioText interface is to improve the literature search experience by better aligning that process with how the literature is read by biologists, and the goal of the study was to determine if this approach provides a better search experience over the standard. Thus, the method used was a qualitative study, and the main measure is self-reported likelihood to use the interface again. (Usage intention has been found in a number of studies to be a good proxy for actual usage behavior [33], [34].) A secondary goal is to determine which details of the interface design are satisfactory and which require improvement.

In this study we choose to evaluate subjective responses rather than standard information retrieval objective measures such as precision and recall or time to find a relevant article. Precision and recall can be valuable measures for comparing ranking algorithms, and may be useful to evaluate after a new interface design has been shown to be acceptable to users. However, experience with the design of novel search interfaces suggests that the most important measure is whether or not people will choose the system over the current standard [32]. Most experimental systems do not pass this test, as evidenced by the fact that the most popular search interfaces have remained relatively unchanged over time (both for Web search and for bioscience literature search). An example of a case in which a qualitatively different style of search interface became the dominant one is found in the tremendous popularity of faceted navigation on e-commerce and digital library web sites [35]. This paradigm was shown to be strongly preferred by study participants [36], and subsequently became accepted and preferred by both designers and users of such sites.

Another popular quantitative measure that can be used for evaluating search interfaces is that of time, in terms of time taken to find relevant documents. That measure is problematic in that it does not account for what is learned about the documents from the search results view. Interfaces that provide more information in the document surrogate tend to require more time per item viewed for users to peruse the richer information [28]. However, as mentioned above, for information-centric queries, it has been shown that richer results listings can be more useful and preferred over short snippets [25], [26]. Nonetheless, a follow up study to this one could measure if the figures helped participants distinguish among relevant documents, or if they were able to save time in the reading of relevant documents by using the summary view.

To conduct the study session, one of the experimenters had a one-on-one session with the participant, lasting approximately 45 minutes (see Table 6). First, the participant read and signed a consent form. Next, the participant was asked general information about their scientific background and their literature searching habits. Next, the experimenter asked the participant for a query, and the experimenter showed the results of running that query in each of the available views. The participant was then instructed to try every view, using the same query or additional queries of their own choice. As they used the system, participants were encouraged to “think aloud,” while the experimenter recorded their actions, reactions and comments. (The “think aloud” protocol is a standard procedure in the evaluation of user interfaces [37].) In the final step, participants were asked to answer a pre-defined set of questions aimed at evaluating each view, and were asked an open-ended question about any additional thoughts or reactions they might have. Participants responses were recorded on paper by the experimenter and then transferred to a spreadsheet.

**Table 6:** Session outline.

Outline of each session (approx 45 min)
**1**. Record basic info of participant (5 min)
Position, area of specialization, how often searches most the literature, favorite literature source, and recent queries.
**2**. Demonstrate briefly the system (5 min)
Introduce participant briefly to the views and their functionalities. Use a query of their choice.
Ask them if they've seen/used the BioText System before and record any views they have on their current experience with the system.
**3**. Observe them using the system and record behavior and comments (10+ min)
Ask participants to try some of their own queries. Encourage them to try all views.
Record queries, comments they make, views they use, and any comments they make or other pertinent information.
**4**. Ask participants, assuming this system contained all articles from all relevant journals, how often would they use each view? (5 min)
Scale: never/rarely/sometimes/frequently
**5**. Ask participants, how often would they use each view per query type? (5+ min)
a. For the queries they used in step 3, ask what kind of information they were looking for:
1. related papers
2. particular information
3. generic background info
4. reviews
5. new developments/findings
6. experimental results
7. new experimental methods
8. other
b. Then for each query ask how often they would use each view.
Scale: never/rarely/sometimes/frequently
**6**. Ask participants to revisit each view and comment what they like and dislike in each (5+ min)
**7**. Ask for general comments/suggestions (5 min)

An ordered list of all the steps completed during each one-on-one session between an experimenter and a participant.

The participants were recruited via flyers placed on campus as well as via personal contacts by the researchers and their associates. The final set of participants consisted of 20 bioscientists (6 graduate students, 6 postdocs, 1 faculty, 7 other). An effort was made to recruit primarily biologists who were not focused on bioinformatics, as people who study information processing tools may have different attitudes towards them than those who focus on the sciences. Most of the participants work in cell or molecular biology, genetics or genomics, biochemistry, evolutionary biology or bioinformatics.

Table 7 shows participants' responses to questions about their fields of interest, how often they search the research literature and which tools they prefer to use. Only one participant had prior experience with a version of the experimental interface. 7 participants said they search the literature daily; 10 said they search one to several times a week and 3 said they search monthly or rarely. All participants use PubMed, and for 16 participants, it is their primary literature searching source. 6 participants also use Google web search, 3 use Google Scholar and another 2 use the Web of Science. Email alerts from journals and myNCBI were also mentioned as well as searching in journal sites, Wikipedia and other specialized sources. Table 8 summarizes the types of queries issued and their frequencies. As found in other studies [38], queries on gene and protein names are the most common. Also common in this collection were queries on author names, species, cell compartments, and methods and analysis techniques. The latter might be more common in our query collection than in other studies due to the ability to search caption text.

**Table 7:** Study participant characteristics.

Status	Area(s) of specialization	Literature search frequency	Preferred search tool(s)
B.S. in biology	evolutionary biology/genomics	few times/week	www.ekt.gr, PubMed, Google scholar
masters student	molecular biology/biochemistry	few times/month	PubMed
PhD student	signalling transduction	daily	PubMed
PhD student	biochemistry	daily	PubMed (Google scholar)
PhD student	bioengineering	few times/week	PubMed, Web of Science
PhD student	biochemistry	few times/week	PubMed (Google)
PhD student (new)	systematics	few times/month	Pathfinder, Google, Google Scholar (Pubmed)
postdoc	cell biology	several times/week	Pubmed, specialized DB
postdoc	cell signalling	several times/week	PubMed
postdoc	molecular/cell biology	daily	PubMed (Google, Textpresso)
postdoc	evolutionary biology/bioinformatics	weekly	PubMed
postdoc	Genetics	daily	PubMed and journal email alerts
postdoc	genomics	daily	PubMed (Web of Science)
professor	genetics	daily	Pubmed (Google)
assistant analyst	molecular/cell biology	rarely	PubMed (Google)
research technician	biochemistry	weekly	Google (PubMed)
research technologist	genetics/chemistry	weekly	Journal sites (PubMed)
associate scientist	cell biology	several times/week	PubMed
senior researcher	bioinformatics	daily	PubMed
retired researcher	bioinformatics	few times/week	PubMed

Characteristics of the study participants, including their professional status and their fields of specialization. The study included 20 bioscientists (6 graduate students, 6 postdocs, 1 faculty, 7 other). Participants were asked to estimate how often they search the research literature and which tools they prefer to use. 7 participants said they search the literature daily; 10 said they search one to several times a week and 3 said they search monthly or rarely.

The final (right-hand) column shows literature search tools used regularly; tools whose use was indicated to be only occasional or rare are shown in parentheses, otherwise no distinction was made about frequency of use. All participants use PubMed, and for 16 participants, it is their primary literature searching source. 6 participants also use Google web search, 3 use Google Scholar and another 2 use the Web of Science. Email alerts from journals and myNCBI were also mentioned as well as searching in journal sites, Wikipedia and other specialized sources (e.g., Saccharomyces Genome Database and Pathfinder).

**Table 8:** Participant queries.

# Query Type	
12	gene/protein name(s)
7	gene/protein name and species name
7	author name(s)
7	method/equipment
4	biological process
4	analysis technique
2	chemical/organic compound
2	species name
2	common DNA sequence
2	analysis technique and species
2	disease
2	cell compartment and protein structure
1	gene/protein subunit
1	gene/protein name and species and cell compartment
1	species name and method
1	species name and author
1	species and cell compartment
1	cell compartment
1	drug
1	plasmid
1	experimental protein
1	biological process and species
1	tissue type and disease

A summary of the kinds of queries that participants issued during the course of the study, at most one of each type of query is counted per participant.
